# Within-ejaculate sperm competition

**DOI:** 10.1098/rstb.2020.0066

**Published:** 2020-10-19

**Authors:** Andreas Sutter, Simone Immler

**Affiliations:** School of Biological Sciences, University of East Anglia, Norwich Research Park, Norwich NR4 7TJ, UK

**Keywords:** haploid selection, meiotic drive, genetic conflict, multi-level selection

## Abstract

Sperm competition was defined by Geoff Parker 50 years ago as the competition between sperm from two or more males over the fertilization of a set of eggs. Since the publication of his seminal paper, sperm competition has developed into a large field of research, and many aspects are still being discovered. One of the relatively poorly understood aspects is the importance of selection and competition among sperm within the ejaculate of a male. The sheer number of sperm present in a male's ejaculate suggests that the competition among sibling sperm produced by the same male may be intense. In this review, we summarize Parker's theoretical models generating predictions about the evolution of sperm traits under the control of the haploid gamete as opposed to the diploid male. We review the existing evidence of within-ejaculate competition from a wide range of fields and taxa. We also discuss the conceptual and practical hurdles we have been facing to study within-ejaculate sperm competition, and how novel technologies may help in addressing some of the currently open questions.

This article is part of the theme issue ‘Fifty years of sperm competition’.

## 1. Sperm in competition

In his landmark paper celebrating its 50th anniversary with this issue, Parker [1] defined *sperm competition* as the competition between sperm from two or more males over the fertilization of eggs. The term *sperm competition*, therefore, by default refers to sperm competition between ejaculates [2]. However, because in the vast majority of species, sperm from one male generally outnumber available eggs, the competition among sibling sperm produced by one male is potentially intense [3,4]. To distinguish between the two forms of sperm competition, we hereafter refer to *between-ejaculate* (between sperm of different males) and *within-ejaculate* (between sperm from one male) competition. While the risk and intensity of between-ejaculate competition vary between mating events and across males and species, within-ejaculate competition may occur during every fertilization event. The role of between-ejaculate sperm competition in the evolution of sperm and male traits is supported by a large body of evidence [5,6], whereas the role and importance of within-ejaculate sperm competition for evolutionary processes is less well documented [7–9]. In this review, we focus on the evolutionary role of within-ejaculate competition. We first summarize Parker's theoretical contribution and then review theoretical arguments and empirical evidence for within-ejaculate competition.

## 2. Parker's models: diploid versus haploid control over sperm phenotype

Among the numerous contributions by Geoff Parker to sperm competition theory (see [10]), two papers, published in parallel, investigated how *diploid* and *haploid* control respectively affect the evolution of sperm characteristics, and how these two scenarios differ [2,3]. Both studies use game theory to identify evolutionary stable strategies (ESS) for sperm number and sperm size, both influencing fertilization success, in the context of between-ejaculate competition. All models share the assumptions that ejaculate costs are the product of sperm number and size, and that variation in sperm size provides diminishing returns for fertilization success. Furthermore, ejaculate costs can either trade-off with achieved matings or be fixed, with a trade-off arising between sperm size and number. The main difference between the two sets of models is the assumption that the evolution of sperm size and number are under the control of the diploid male [2] or under the control of the haploid sperm [3].

The ESS differs substantially between the two sets of models. Under diploid male control, sperm numbers are predicted to increase with the risk of between-ejaculate sperm competition, whereas size shows no effect, unless density-dependence or survival benefits for larger sperm are invoked [2]. When sperm phenotypes are under haploid gametic control, the predicted outcome depends on whether the cost of the mutation favouring the mutant sperm is paid by the male, by sibling sperm carrying the alternative allele, or by sibling sperm carrying the same mutation [3]. Where costs are assumed by the male, size and number mutations (i.e. by diverting resources to increasing sperm size or to increasing rate of cell division and hence sperm number) are predicted to escalate at the expense of achieved number of matings. If the cost is paid by sibling non-mutant sperm, size or number mutations can spread under a size–number trade-off, while mutations that are costly to sibling mutant sperm carrying the same allele do not spread.

**Figure 1.**
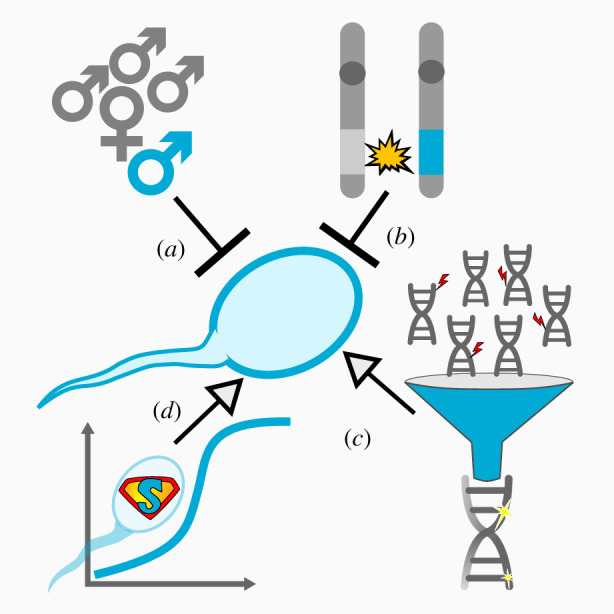
Factors that are expected to hinder or favour within-ejaculate sperm competition. (*a*) Between-ejaculate sperm competition is predicted to reduce the importance of within-ejaculate sperm competition. (*b*) While mutant alleles with a haploid advantage may favour within-ejaculate competition, alternative alleles paying the cost of the mutant allele should suppress within-ejaculate sperm competition. If mutant alleles favoured in within-ejaculate sperm competition have deleterious effects on diploid fitness, the entire diploid genome is under selection to evolve a resistance mechanism to suppress the mutant allele. (*c*) If efficient purifying selection via haploid selection is possible, selection should favour within-ejaculate sperm competition. (*d*) A similar situation occurs if mutations are beneficial for within-ejaculate sperm competition and diploid fitness. Such alleles are expected to quickly sweep to fixation and will be hard to trace. (Online version in colour.)

One intuitive prediction resulting from the conflict between a male and its sperm is that within-ejaculate competition in species with high risk of sperm competition should be minimized, owing to the potential costs to the male (figure 1*a*). However, Parker & Begon [3] showed that even under maximum risk of between-ejaculate sperm competition, conflicts between male and sperm do not disappear [3]. Indeed, theoretical models for the evolution of ‘soldier sperm’ attacking a rival male's sperm by sacrificing their own fertilization ability in favour of sibling sperm show that these can only evolve if the control lies with the diploid male [11]. A more recent model predicted that alleles favoured in within-ejaculate competition can spread rapidly if they are neutral (or beneficial) with respect to diploid fitness [12]. Similarly, another model confirmed that haploid selection is maintained even under scenarios of sperm competition, if selection on haploid gametes results in the efficient removal of deleterious mutations [13].

## 3. Within-ejaculate competition driving sperm evolution

A male shares 50% of its alleles with all its sperm carrying a full set of haploid chromosomes. Sibling sperm are on average also 50% related to one another but this may vary depending on the rate of segregation, recombination and the heterozygosity of an organism. This situation could be compared to scenarios of parent–offspring conflict, where individual offspring are selected to be selfish at the cost of parental fitness [14,15]. Sperm traits that have been hypothesized to mediate possible conflicts in favour of the diploid male include a densely re-packaged DNA and suppression of post-meiotic transcription, cytoplasmic bridges linking haploid spermatids with each other for efficient sharing of transcripts, and control of haploid gametes through diploid-expressed RNA or seminal fluid [16–18]. For sperm traits to evolve through within-ejaculate selection, three general criteria for evolution need to be met: (i) sperm need to exhibit phenotypic variation; (ii) sperm phenotypes must be heritable; and (iii) sperm phenotypes need to affect fitness [19]. We only briefly discuss evidence for each of these, as all three have been discussed earlier in extensive reviews (e.g. [8,9,20]).

Phenotypic variation among sibling sperm is well documented, but whether this variation arises for accidental or adaptive reasons is still not fully understood [20–22]. Potential, non-mutually exclusive explanations for phenotypic variation include sperm production errors (e.g. [23]), strategic variation for bet-hedging [24], distinct casts of sperm phenotypes [11,25], and manifestation of haploid interests [3,9]. Observed patterns are often compatible with several of these hypotheses. For example, the observation that within-ejaculate phenotypic variation correlates negatively with the level of sperm competition (e.g. [26,27]; but see [28]) could be explained by stabilizing selection on optimal sperm phenotypes under increased risk of sperm competition [29], but also by a reduction of the haploid–diploid conflict with increasing importance of between-ejaculate competition in species with high sperm competition risk ([3]; see [30] for a rare exception).

**Figure 2.**
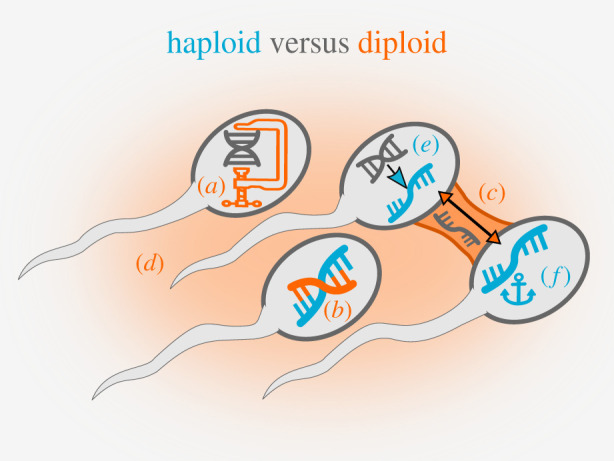
Biological mechanisms facilitating versus suppressing within-ejaculate sperm competition. Schematic of conflict between haploid sperm and the diploid organism over control of sperm phenotype. Mechanisms by which sperm may facilitate (blue) and the diploid organism may hamper (orange) haploid control, respectively, are shown. The diploid organism may attempt to silence haploid gene expression through (*a*) DNA condensation or (*b*) RNA interference, and may eliminate differences between sperm through (*c*) sharing of haploid-expressed RNAs and proteins via cytoplasmic bridges, or through (*d*) control over sperm phenotype by seminal fluid. Sperm may attempt (*e*) haploid transcription/translation, and (*f*) haploid retention of RNA and proteins to avoid homogenization among sibling sperm. (Online version in colour.)

In order for phenotypic variation to be heritable, sperm phenotypes need to at least partially reflect the haploid sperm genotype (figure 2). It was long thought that genome condensation in developing sperm would largely silence gene expression (e.g. [31]), and that cytoplasmic bridges between spermatids would essentially homogenize any potential remaining differences [32]. The very fact that sperm are so small may be related to avoiding selfish genetic (cytoplasmic) elements acting in sperm [33], and the evolution of other aspects of spermatogenesis may have been fuelled by intragenomic conflict with selfish genetic elements [34]. Nevertheless, there is now ample evidence for post-meiotic transcription [7,32,35,36] and many transcripts are not equally shared via cytoplasmic bridges [37–40]. Ways for males to control the effects of haploid-expressed genes and prevent within-ejaculate competition are for example by provisioning sperm with diploid-derived RNA [17] or by affecting sperm via the composition of the seminal fluid [41,42].

Finally, even if sperm are able to express their genotype, this expression needs to result in a phenotypic difference that influences their chance of winning fertilizations. Although it is conceivable and intuitive that different phenotypes would have different chances of fertilizing ova, this connection is not always explicitly established. Empirical evidence for within-ejaculate competition with fitness consequences and thus evolutionary potential comes from some meiotic drivers [37,39,43]. Outside of these (perhaps extreme) examples, indications that within-ejaculate competition has evolutionary potential comes from studies linking within-ejaculate sperm selection to offspring fitness [44–46], though the underlying mechanisms remain somewhat elusive.

## 4. Potential costs and benefits of within-ejaculate competition

In most species, sperm are produced in vast numbers, but only very few of them end up fertilizing eggs, potentially resulting in strong selection for the ‘best’ sperm (e.g. [12,47]). Novel genotypes can be generated through *de novo* mutations, recombination and segregation events, and natural selection for the best sperm may act in two ways: purifying selection removing deleterious mutations and genotypes (figure 1*c*), and positive selection for optimal genotypes and beneficial mutations [47] (reviewed in [8,9]; figure 1*d*). While in a diploid genome, recessive alleles may hide from selection [48,49] and any beneficial or deleterious alleles expressed in a haploid genome will be exposed to selection, rendering haploid selection much more efficient. If a haplotype's performance in gametic selection is correlated with its fitness effects in the diploid phase, selection at the haploid gametic stage could offer a cheap and efficient way of trying out new allelic combinations [9].

As mentioned above, under gametic control over sperm traits, mutant sperm gain within-ejaculate competitiveness either at the expense of the diploid male, their sibling sperm with an alternative allele, or their sibling sperm with the same mutant allele [3]. Empirical data from sperm competitiveness of males with meiotic drivers suggest that a combination of all three scenarios can occur [50,51]. When mutant sperm gain a fitness advantage at the expense of sibling sperm carrying an alternative allele, intra-locus conflict will arise (figure 1*b*). Moreover, if the haploid mutant allele has a deleterious effect in diploids, the conflict can extend to the rest of the genome, and selection on the diploid genome should favour suppression of the selfish mutant allele [52]. Thus, if within-ejaculate competition is costly for the diploid male, lineages that can silence this competition are expected to outcompete lineages that do not [34,52,53]. However, the efficiency of haploid selection allows alleles with deleterious effects in the diploid organism to remain in a population [54]. In fact, even alleles that are recessive lethal to the diploid organism can increase in frequency if their effects are sufficiently beneficial for within-ejaculate competition [55]. However, because these alleles are recessive lethal, they cannot go to fixation and a stable polymorphism prevents the population from reaching its fitness maximum [56].

## 5. Evidence for and against within-ejaculate sperm competition

While evidence for evolution through between-ejaculate competition has been shown across taxa in a large body of experimental and comparative studies [5], the evidence for evolution through within-ejaculate competition is much scarcer. Part of the reason for the paucity of studies is the technical difficulty of showing a process occurring between cells during an often very limited amount of time. In addition, such competition can often only be monitored inside the female reproductive tract or an aquatic environment, making the tracking of individual sperm virtually impossible. An additional reason is the aforementioned long-standing assumption that genetic differences among haploid sperm contribute little if anything to the phenotypic variation (reviewed in [7]). This view has recently been challenged as the evidence for gene expression at the post-meiotic haploid stage is steadily increasing (e.g. [40,43,57]; for reviews see [7–9]). While haploid gene expression is not the only way haploid selection among sperm can operate, it certainly increases the opportunity for evolution through within-ejaculate competition.

The most convincing evidence for within-ejaculate competition comes from studies in a range of plants. Haploid gene expression in pollen is well established, and experimental evidence suggests that competition among pollen from the same male improves the fitness of the resulting seedlings [58]. In addition, two studies in the grand shepherd's purse *Capsella grandiflora* (an extreme outcrossing species) and the thale cress *Arabidopsis thaliana* (an extreme selfing species) showed increased levels of purifying and positive selection among genes expressed at the haploid stage [59,60]. The fact that a similar genomic signature is found in species with very contrasting levels of outcrossing suggests that the outcome of haploid selection may be aligned with diploid fitness interests in these species. In animals, several recent studies have provided evidence for selection and competition among haploid sperm. In the zebrafish *Danio rerio*, pools of longer-lived sperm exhibited allelic differences across the entire genome compared to shorter-lived and immotile sperm [45]. Similarly, a link between marker alleles and sperm phenotypes has been reported in a male hybrid between two *Astyanax* cavefish [61]. In that study, sperm were exposed to a dye challenge, resulting in the grouping of sperm phenotypes sharing similar allelic contents. In mammals, the most direct evidence for a link between sperm genotype and sperm phenotype comes from studies in the house mouse *Mus musculus*, where X‐ and Y‐bearing sperm show differences in motility that are not driven by the difference in physical size [43]—a factor that has been suggested to explain differences in motility between human X‐ and Y-sperm [62]. Sperm sexing based on membrane proteins in mice has been proposed as an efficient mechanism to determine offspring sex in domestic cattle [63], though it is questionable whether this would translate from *in vitro* into *in vivo* applications [64]. In domestic bull (*Bos taurus*), X- and Y-sperm differ by nine nuclear DNA coded proteins [65]. The different survival of X- and Y-sperm in the female reproductive tract of mammals including humans has been suggested several times, but these observations are currently still anecdotal. The recent findings of a wide range of genes showing biased gene expression across haploid spermatids in house mice and the cynomolgus primate *Macaca fasciculari*s, with the same genes showing signs of directional selection in primate and human populations [40], suggest that a rather large number of genes could actually be involved in determining sperm phenotypes. Again, the function of these genes and their effect on sperm phenotype are currently unclear.

Some indirect evidence for the potential of within-ejaculate competition may come from the fact that many species with a high risk of sperm competition produce dimorphic sperm, which vary not only in their morphology and size, but also their genetic content [21,24]. Often one of the two sperm morphotypes shows a partial or complete lack of DNA (apyrene sperm), rendering them incapable of fertilizing eggs [66]. Apyrene sperm appear to have the sole purpose of aiding sperm competition processes by occupying space inside the female sperm storage organs, and/or of protecting sibling sperm from the hostile environment inside the female reproductive tract [67]. The lack of DNA in apyrene sperm results in the effective removal of any genetic conflict with their eupyrene sibling sperm, and could be seen as an efficient way to allow for sperm cooperation. However, sperm cooperation has also been suggested in other taxa not exhibiting any obvious sperm dimorphism. In the New World opossum *Didelphis virginiana* for example, two sperm joined at their heads are necessary to reach the site of fertilization, but only one sperm will be able to fertilize the egg as the other one has to undergo an acrosome reaction to separate from its sibling sperm [68]. A similar process of acrosome reaction is necessary for sperm in a ‘train’ to dislocate from each other in the European wood mouse *Apodemus sylvaticus* [69]. A remaining question at this point is whether sperm that undergo acrosome reaction differ genetically from those that get to fertilize the egg, or whether this is a process of pure chance. More generally, the question about whether these observations are a form of cooperation in the evolutionary sense remains controversial [70–73]. While sperm can preferentially cooperate with sibling sperm from the same male when mixed with a competitor male's sperm in the deer mouse *Peromyscus maniculatus* [72], how the roles are divided within an ejaculate is currently unknown [74]. General predictions are that cooperation among sperm could dynamically arise through male enforcement and be eroded by sperm selfishness [11,17,25,75].

As discussed above, part of the dearth of evidence for within-ejaculate sperm competition may have been caused by the lack of technologies, which are now becoming available. Another reason for the scarcity of evidence could be that a *de novo* mutation that is beneficial for the haploid phase would go to fixation relatively rapidly ([12]; figure 1*d*). This is particularly true if it has no effects or a positive effect at the diploid life stage. The detection of such mutations would be difficult, as these would have to be tracked before fixation. The only way to maintain a genetic polymorphism is if such haploid-beneficial mutations have a negative, partly recessive effect inducing fitness cost to the diploid phase, which results in balancing selection [54]. Such situations are well-described in meiotic drivers, where selfish benefits in the (typically male) haploid phase are counterbalanced by costs in the diploid phase ([56]; see also [34]).

Finally, it is possible that some sperm traits are under haploid control while others are under diploid control. The evidence for diploid control over morphological sperm traits and sperm total length in particular (usually largely determined by the length of the flagellum) is convincing. An explicit test of diploid versus haploid control over the evolution of sperm length was performed in a study on *Drosophila* fruit fly lines that had been selected for long and short sperm, respectively [21]. F1 crosses between these lines were performed with the prediction that if sperm length was at least partially determined by the haploid genotype, crosses between the lines should show increased variation in sperm length compared to the two parental strains. However, the offspring from crosses between the two lines showed intermediate lengths of sperm and no increased variation compared to the parental lines. By contrast, a recent study using a similar approach of crossing two *Astyanax* cavefish species to generate increased heterozygosity in the F1 offspring reported increased variation in sperm swimming velocity [76]. Many possible biological mechanisms can explain the divergent observations between these two studies, and we can currently only speculate as to which are true.

## 6. The future of within-ejaculate sperm competition

The past few years have provided some exciting new insights into the role and importance of within-ejaculate competition. However, we are only at the beginning of understanding what is really happening at this stage of the life cycle, and key questions currently remain unanswered. Based on the topics we reviewed in the previous sections, we discuss some of the currently open questions and how it may be possible to address them.

The first set of questions evolves around identifying the ‘best’ sperm in an ejaculate: is there a ‘best’ sperm and if so, which one is it? Which traits contribute to the success of a sperm in within-ejaculate sperm competition? Do these depend on environmental conditions? These questions are difficult to answer at the moment and opinions are divided. Evidence suggesting that the differences among sperm/pollen in how they fertilize eggs are at least partly genetically determined is quite strong [45,58]. However, the exact genomic mechanisms are currently not known. The finding of increased purifying selection in haploid-expressed genes in flowering plants and mammals suggests that competition and selection among sibling sperm may serve as a potential quality check allowing the separation of the ‘wheat from the chaff’. It appears that in both pollen and sperm, physiological performance rather than morphology ultimately determines differences among sibling gametes. A methodological part of the challenge is understanding which sperm characteristics are important for fertilization potential, particularly in internal fertilizers. Morphological variation in sperm length or shape are relatively easy to measure, and can be a good proxy for fertilization success, at least when comparing between males (for reviews see [77,78]). The current literature shows a bias towards detailed studies of morphology, but more recent developments for example in microfluidics [79], single-cell sequencing [80] and the ‘omics revolution [81] allow more detailed assays of individual sperm performance *in vitro* and *in vivo*, and a comparison of the two (e.g. [82]; reviewed in [83]). A further possible challenge is that the traits under selection may not always be the same if environmental conditions vary during fertilization—which they often do [84]. Moreover, the fertilization environment is partly determined by females, arguably more so in internal fertilizers [85]. In any case, heterogeneity in environments and coevolutionary dynamics between the sexes make understanding the complexity of sperm evolution a formidable challenge [84].

A second question is about whether variation—both genetic and phenotypic—among sibling sperm is systematic as opposed to arising from simple ‘production errors’. Understanding the role of purifying and directional selection, as well as understanding which sperm traits are under diploid and which are under haploid control, are the future challenges we are facing. Technologies such as single-cell sequencing and more generally single-cell ‘omics will help in addressing these questions.

A third question is about the methods and species that are best suited for the study of within-ejaculate sperm competition. The ability to generate a natural fertilization environment *in vitro* is key to understanding the biologically relevant sperm traits under selection [78,83]. An alternative route is to employ ever-improving technology such as micro-filming *in situ*, allowing the tracking of sperm within the female reproductive tract [86]. Alternatively, we can use sequencing and genotyping technologies to assess genetic similarities and differences among offspring sired by varying sperm phenotypes selected for specific traits. In this case, species producing large numbers of offspring may be beneficial, but this can be alleviated if offspring from many families are genotyped.

## Data accessibility

This article has no additional data.

## References

[1] Parker GA 1970 Sperm competition and its evolutionary consequences in the insects. Biol. Rev. 45, 525–567. (10.1111/j.1469-185X.1970.tb01176.x)

[2] Parker GA 1993 Sperm competition games: sperm size and sperm number under adult control. Proc. R. Soc. Lond. B 253, 245–254. (10.1098/rspb.1993.0110) 8234363

[3] Parker GA, Begon ME 1993 Sperm competition games: sperm size and number under gametic control. Proc. R. Soc. Lond. B 253, 255–262. (10.1098/rspb.1993.0111) 8234364

[4] Haig D, Bergstrom CT 1995 Multiple mating, sperm competition and meiotic drive. J. Evol. Biol. 8, 265–282. (10.1046/j.1420-9101.1995.8030265.x)

[5] Birkhead TR, Møller AP (eds) 1998 Sperm competition and sexual selection. London, UK: Academic Press.

[6] Birkhead TR, Hosken DJ, Pitnick S (eds) 2009 Sperm biology: an evolutionary perspective. Burlington, MA: Elsevier Ltd.

[7] Joseph S, Kirkpatrick M 2004 Haploid selection in animals. Trends Ecol. Evol. 19, 592–597. (10.1016/j.tree.2004.08.004)

[8] Immler S, Otto SP 2018 The evolutionary consequences of selection at the haploid gametic stage. Am. Nat. 192, 241–249. (10.1086/698483) 30016160

[9] Immler S 2019 Haploid selection in ‘diploid’ organisms. Annu. Rev. Ecol. Evol. Syst. 50, 219–236. (10.1146/annurev-ecolsys-110218-024709)

[10] Parker GA 2020 Conceptual developments in sperm competition: a very brief synopsis. Phil. Trans. R. Soc. B 375, 20200061 (10.1098/rstb.2020.0061) 33070727 PMC7661437

[11] Kura T, Nakashima Y 2000 Conditions for the evolution of soldier sperm classes. Evolution 54, 72–80. (10.1111/j.0014-3820.2000.tb00009.x) 10937185

[12] Ezawa K, Innan H 2013 Competition between the sperm of a single male can increase the evolutionary rate of haploid expressed genes. Genetics 194, 709–719. (10.1534/genetics.113.152066) 23666936 PMC3697975

[13] Otto SP, Scott MF, Immler S 2015 Evolution of haploid selection in predominantly diploid organisms. Proc. Natl Acad. Sci. USA 112, 15 952–15 957. (10.1073/pnas.1512004112) PMC4703000 26669442

[14] Trivers RL 1974 Parent–offspring conflict. Am. Zool. 14, 249–264. (10.1093/icb/14.1.249)

[15] Godfray H 1995 Evolutionary theory of parent–offspring conflict. Nature 376, 133–138. (10.1038/376133a0) 7603563

[16] Frank SA 1995 Mutual policing and repression of competition in the evolution of cooperative groups. Nature 377, 520–522. (10.1038/377520a0) 7566147

[17] Hosken DJ, Hodgson DJ 2014 Why do sperm carry RNA? Relatedness, conflict, and control. Trends Ecol. Evol. 29, 451–455. (10.1016/j.tree.2014.05.006) 24916312

[18] Ågren JA, Davies NG, Foster KR 2019 Enforcement is central to the evolution of cooperation. Nat. Ecol. Evol. 3, 1018–1029. (10.1038/s41559-019-0907-1) 31239554

[19] Lewontin R 1970 The units of selection. Annu. Rev. Ecol. Syst. 1, 1–18. (10.1146/annurev.es.01.110170.000245)

[20] Holt WV, Van Look KJW 2004 Concepts in sperm heterogeneity, sperm selection and sperm competition as biological foundations for laboratory test of semen quality. Reproduction 127, 527–535. (10.1530/rep.1.00134) 15129008

[21] Pitnick S, Hosken D, Birkhead T 2009 Sperm morphological diversity. In Sperm biology: an evolutionary perspective (eds Birkhead T, Hosken D, Pitnick S), pp. 69–149. Burlington, MA: Elsevier Ltd.

[22] Higginson DM, Pitnick S 2011 Evolution of intra-ejaculate sperm interactions: do sperm cooperate? Biol. Rev. Camb. Philos. Soc. 86, 249–270. (10.1111/j.1469-185X.2010.00147.x) 20608927

[23] Stewart KA, Wang R, Montgomerie R 2016 Extensive variation in sperm morphology in a frog with no sperm competition. BMC Evol. Biol. 16, 29 (10.1186/s12862-016-0601-8) 26832366 PMC4735968

[24] Till-Bottraud I, Joly D, Lachaise D, Snook RR 2005 Pollen and sperm heteromorphism: convergence across kingdoms? J. Evol. Biol. 18, 1–18. (10.1111/j.1420-9101.2004.00789.x) 15669956

[25] Pizzari T, Foster KR 2008 Sperm sociality: cooperation, altruism, and spite. PLoS Biol. 6, e130 (10.1371/journal.pbio.0060130) 18507504 PMC2430914

[26] Immler S, Calhim S, Birkhead TR 2008 Increased postcopulatory sexual selection reduces the intramale variation in sperm design. Evolution 62, 1538–1543. (10.1111/j.1558-5646.2008.00393.x) 18384656

[27] Lifjeld JT, Laskemoen T, Kleven O, Albrecht T, Robertson RJ 2010 Sperm length variation as a predictor of extrapair paternity in passerine birds. PLoS ONE 5, 1–8. (10.1371/journal.pone.0013456) PMC2956655 20976147

[28] Sharma MD, Minder AM, Hosken DJ 2013 No association between sperm competition and sperm length variation across dung flies (Scathophagidae). J. Evol. Biol. 26, 2341–2349. (10.1111/jeb.12232) 24016061

[29] Bernasconi G, Hellriegel B 2005 Fertilization competence and sperm size variation in sperm-heteromorphic insects. Evol. Ecol. 19, 45–54. (10.1007/s10682-004-7594-2)

[30] Fitzpatrick JL, Baer B 2011 Polyandry reduces sperm length variation in social insects. Evolution 65, 3006–3012. (10.1111/j.1558-5646.2011.01343.x) 21967440

[31] Steger K 1999 Transcriptional and translational regulation of gene expression in haploid spermatids. Anat. Embryol. (Berl). 199, 471–487. (10.1007/s004290050245) 10350128

[32] Dadoune JP, Siffroi JP, Alfonsi MF 2004 Transcription in haploid male germ cells. Int. Rev. Cytol. 237, 1–56. (10.1016/S0074-7696(04)37001-4) 15380665

[33] Randerson JP, Hurst LD 1999 Small sperm, uniparental inheritance and selfish cytoplasmic elements: a comparison of two models. J. Evol. Biol. 12, 1110–1124. (10.1046/j.1420-9101.1999.00112.x)

[34] Verspoor RL, Price TAR, Wedell N 2020 Selfish genetic elements and male fertility. Phil. Trans. R. Soc. B 375, 20200067 (10.1098/rstb.2020.0067) 33070738 PMC7661447

[35] Vibranovski MD, Chalopin DS, Lopes HF, Long M, Karr TL 2010 Direct evidence for postmeiotic transcription during *Drosophila melanogaster* spermatogenesis. Genetics 186, 431–433. (10.1534/genetics.110.118919) 20610406 PMC2940308

[36] Ren X, Chen X, Wang Z, Wang D 2017 Is transcription in sperm stationary or dynamic? J. Reprod. Dev. 63, 439–443. (10.1262/jrd.2016-093) 28845020 PMC5649092

[37] Zheng Y, Deng X, Martin-DeLeon PA 2001 Lack of sharing of Spam1 (Ph-20) among mouse spermatids and transmission ratio distortion. Biol. Reprod. 64, 1730–1738. (10.1095/biolreprod64.6.1730) 11369602

[38] Ventelä S, Toppari J, Parvinen M 2003 Intercellular organelle traffic through cytoplasmic bridges in early spermatids of the rat: mechanisms of haploid gene product sharing. Mol. Biol. Cell 14, 2768–2780. (10.1091/mbc.e02-10-0647) 12857863 PMC165675

[39] Véron N, Bauer H, Weiße A 2009 Retention of gene products in syncytial spermatids promotes non-Mendelian inheritance as revealed by the *t* *complex responder*. Genes Dev. 23, 2705–2710. (10.1101/gad.553009) 19952105 PMC2788329

[40] Bhutani K et al 2019 Widespread haploid-biased gene expression in mammalian spermatogenesis associated with frequent selective sweeps and evolutionary conflict. bioRxiv 846253 (10.1101/846253)

[41] Pizzari T, Parker GA 2009 Sperm competition and sperm phenotype. In Sperm biology: an evolutionary perspective (eds Birkhead TR, Hosken DJ, Pitnick S), pp. 207–245. Burlington, MA: Elsevier Ltd.

[42] Perry JC, Sirot L, Wigby S 2013 The seminal symphony: how to compose an ejaculate. Trends Ecol. Evol. 28, 414–422. (10.1016/j.tree.2013.03.005) 23582755 PMC4974483

[43] Rathje CC et al. 2019 Differential sperm motility mediates the sex ratio drive shaping mouse sex chromosome evolution. Curr. Biol. 29, 3692–3698. (10.1016/j.cub.2019.09.031) 31630954 PMC6839398

[44] Immler S, Hotzy C, Alavioon G, Petersson E, Arnqvist G 2014 Sperm variation within a single ejaculate affects offspring development in Atlantic salmon. Biol. Lett. 10, 20131040 (10.1098/rsbl.2013.1040) 24522632 PMC3949374

[45] Alavioon G et al. 2017 Haploid selection within a single ejaculate increases offspring fitness. Proc. Natl Acad. Sci. USA 114, 8053–8058. (10.1073/pnas.1705601114) 28698378 PMC5544320

[46] Pérez-Cerezales S et al. 2018 Sperm selection by thermotaxis improves ICSI outcome in mice. Sci. Rep. 8, 2902 (10.1038/s41598-018-21335-8) 29440764 PMC5811574

[47] Manning J, Chamberlain A 1994 Sib competition and sperm competitiveness: an answer to ‘why so many sperms?’ and the recombination/sperm number correlation. Proc. R. Soc. Lond. B 256, 177–182. (10.1098/rspb.1994.0067) 8029242

[48] Crow J, Kimura M 1970 An introduction to population genetics theory. Manhattan, New York: Harper & Row.

[49] Orr HA, Otto SP 1994 Does diploidy increase the rate of adaptation? Genetics 136, 1475–1480. 8013920 10.1093/genetics/136.4.1475 PMC1205926

[50] Price TAR, Bretman AJ, Avent TD, Snook RR, Hurst GDD, Wedell N 2008 Sex ratio distorter reduces sperm competitive ability in an insect. Evolution 62, 1644–1652. (10.1111/j.1558-5646.2008.00386.x) 18373627

[51] Sutter A, Lindholm AK 2015 Detrimental effects of an autosomal selfish genetic element on sperm competitiveness in house mice. Proc. R. Soc. B 282, 20150974 (10.1098/rspb.2015.0974) PMC4528557 26136452

[52] Leigh EG 1977 How does selection reconcile individual advantage with the good of the group? Proc. Natl Acad. Sci. USA 74, 4542–4546. (10.1073/pnas.74.10.4542) 16592453 PMC431981

[53] Prout T, Bundgaard J, Bryant S 1973 Population genetics of modifiers of meiotic drive I. The solution of a special case and some general implications. Theor. Popul. Biol. 4, 446–465. (10.1016/0040-5809(73)90020-8) 4779109

[54] Immler S, Arnqvist G, Otto S 2012 Ploidally antagonistic selection maintains stable genetic polymorphism. Evolution 66, 55–65. (10.1111/j.1558-5646.2011.01399.x) 22220864

[55] Bruck D 1957 Male segregation ratio advantage as a factor in maintaining lethal alleles in wild populations of house mice. Proc. Natl Acad. Sci. USA 43, 152–158. (10.1073/pnas.43.1.152) 16589989 PMC528400

[56] Lindholm AK et al. 2016 The ecology and evolutionary dynamics of meiotic drive. Trends Ecol. Evol. 31, 315–326. (10.1016/j.tree.2016.02.001) 26920473

[57] Raices JB, Otto PA, Vibranovski MD 2019 Haploid selection drives new gene male germline expression. Genome Res. 29, 1115–1122. (10.1101/gr.238824.118) 31221725 PMC6633266

[58] Niesenbaum RA 1999 The effects of pollen load size and donor diversity on pollen performance, selective abortion, and progeny vigor in *Mirabilis jalapa* (Nyctaginaceae). Am. J. Bot. 86, 261–268. (10.2307/2656941) 21680363

[59] Arunkumar R, Josephs EB, Williamson RJ, Wright SI 2013 Pollen-specific, but not sperm-specific, genes show stronger purifying selection and higher rates of positive selection than sporophytic genes in *Capsella grandiflora*. Mol. Biol. Evol. 30, 2475–2486. (10.1093/molbev/mst149) 23997108

[60] Gossmann TI, Schmid MW, Grossniklaus U, Schmid KJ 2014 Selection-driven evolution of sex-biased genes is consistent with sexual selection in *Arabidopsis thaliana*. Mol. Biol. Evol. 31, 574–583. (10.1093/molbev/mst226) 24273323

[61] Borowsky R, Luk A, He X, Kim RS 2018 Unique sperm haplotypes are associated with phenotypically different sperm subpopulations in *Astyanax* fish. BMC Biol. 16, 1–11. (10.1186/s12915-018-0538-z) 29973198 PMC6032774

[62] Cui K 1997 Size differences between human X and Y spermatozoa and prefertilization diagnosis. Mol. Hum. Reprod. 3, 61–67. (10.1093/molehr/3.1.61) 9239709

[63] Umehara T, Tsujita N, Shimada M 2019 Activation of Toll-like receptor 7/8 encoded by the X chromosome alters sperm motility and provides a novel simple technology for sexing sperm. PLoS Biol. 17, e3000398 (10.1371/journal.pbio.3000398) 31408454 PMC6691984

[64] Navarro-Costa PA, Molaro A, Misra CS, Meiklejohn CD, Ellis PJ 2020 Sex and suicide: the curious case of Toll-like receptors. PLoS Biol. 18, e3000663 (10.1371/journal.pbio.3000663) 32203540 PMC7117759

[65] Scott C et al. 2018 Proteomic profile of sex-sorted bull sperm evaluated by SWATH-MS analysis. Anim. Reprod. Sci. 198, 121–128. (10.1016/j.anireprosci.2018.09.010) 30274742

[66] Snook RR, Karr TL 1998 Only long sperm are fertilization-competent in six sperm-heteromorphic *Drosophila* species. Curr. Biol. 8, 291–294. (10.1016/S0960-9822(98)70112-5) 9501071

[67] Holman L, Snook RR 2008 A sterile sperm caste protects brother fertile sperm from female-mediated death in *Drosophila pseudoobscura*. Curr. Biol. 18, 292–296. (10.1016/j.cub.2008.01.048) 18291649

[68] Rodger JC, Bedford JM 1982 Separation of sperm pairs and sperm–egg interaction in the opossum, *Didelphis virginiana*. Reproduction 64, 171–179. (10.1530/jrf.0.0640171) 7054492

[69] Moore H, Dvořáková K., Jenkins N, Breed W 2002 Exceptional sperm cooperation in the wood mouse. Nature 418, 174–177. (10.1038/nature00832) 12110888

[70] Immler S, Moore HDM, Breed WG, Birkhead TR 2007 By hook or by crook? Morphometry, competition and cooperation in rodent sperm. PLoS ONE 2, e170 (10.1371/journal.pone.0000170) 17245446 PMC1764683

[71] Firman RC, Simmons LW 2009 Sperm competition and the evolution of the sperm hook in house mice. J. Evol. Biol. 22, 2505–2511. (10.1111/j.1420-9101.2009.01867.x) 19878408

[72] Fisher HS, Hoekstra HE 2010 Competition drives cooperation among closely related sperm of deer mice. Nature 463, 801–803. (10.1038/nature08736) 20090679 PMC2824558

[73] Varea-Sanchez M, Tourmente M, Bastir M, Roldan ERS 2016 Unraveling the sperm bauplan: relationships between sperm head morphology and sperm function in rodents. Biol. Reprod. 95, 25 (10.1095/biolreprod.115.138008) 27281707

[74] Moore T, Moore HD 2002 Marsupial sperm pairing: a case of ‘sticky’ green beards? Trends Ecol. Evol. 17, 112–113. (10.1016/S0169-5347(01)02425-9)

[75] Immler S 2008 Sperm competition and sperm cooperation: the potential role of diploid and haploid expression. Reproduction 135, 275–283. (10.1530/REP-07-0482) 18299420

[76] Borowsky R, Luk A, Kim RS 2019 Sperm swimming behaviors are correlated with sperm haploid genetic variability in the Mexican tetra, *Astyanax mexicanus*. PLoS ONE 14, e0218538 (10.1371/journal.pone.0218538) 31242252 PMC6594619

[77] Snook RR 2005 Sperm in competition: not playing by the numbers. Trends Ecol. Evol. 20, 46–53. (10.1016/j.tree.2004.10.011) 16701340

[78] Lüpold S, Pitnick S 2018 Sperm form and function: what do we know about the role of sexual selection? Reproduction 155, R229–R243. (10.1530/REP-17-0536) 29459400

[79] Knowlton SM, Sadasivam M, Tasoglu S 2015 Microfluidics for sperm research. Trends Biotechnol. 33, 221–229. (10.1016/j.tibtech.2015.01.005) 25798781

[80] Wang J, Fan HC, Behr B, Quake SR 2012 Genome-wide single-cell analysis of recombination activity and de novo mutation rates in human sperm. Cell 150, 402–412. (10.1016/j.cell.2012.06.030) 22817899 PMC3525523

[81] Baker MA 2011 The ‘omics revolution and our understanding of sperm cell biology. Asian J. Androl. 13, 6–10. (10.1038/aja.2010.62) 20972449 PMC3739389

[82] Holt WV, Hernandez M, Warrell L, Satake N 2010 The long and the short of sperm selection *in vitro* and *in vivo*: swim-up techniques select for the longer and faster swimming mammalian sperm. J. Evol. Biol. 23, 598–608. (10.1111/j.1420-9101.2010.01935.x) 20487132

[83] Hook KA, Fisher HS 2020 Methodological considerations for examining the relationship between sperm morphology and motility. Mol. Reprod. Dev. 87, 633–649. (10.1002/mrd.23346) 32415812 PMC7329573

[84] Reinhardt K, Dobler R, Abbott J 2015 An ecology of sperm: sperm diversification by natural selection. Annu. Rev. Ecol. Evol. Syst. 46, 435–459. (10.1146/annurev-ecolsys-120213-091611)

[85] Birkhead TR, Møller AP, Sutherland WJ 1993 Why do females make it so difficult for males to fertilize their eggs? J. Theor. Biol. 161, 51–60. (10.1006/jtbi.1993.1039)

[86] Manier MK, Belote JM, Berben KS, Novikov D, Stuart WT, Pitnick S 2010 Resolving mechanisms of competitive fertilization success in *Drosophila melanogaster*. Science 328, 354–357. (10.1126/science.1187096) 20299550

